# Comparative effectiveness of short-term psychodynamic psychotherapy and cognitive behavioral therapy for major depression in psychiatric outpatient clinics: a randomized controlled trial

**DOI:** 10.1186/s12888-025-06544-6

**Published:** 2025-02-11

**Authors:** Anders Malkomsen, Theresa Wilberg, Bente Bull-Hansen, Toril Dammen, Julie Horgen Evensen, Benjamin Hummelen, André Løvgren, Kåre Osnes, Randi Ulberg, Jan Ivar Røssberg

**Affiliations:** 1 Division of Mental Health and Addiction, Oslo University Hospital, Oslo, Norway; 2 Institute of Clinical Medicine, University of Oslo, Oslo, Norway; 3 Diakonhjemmet Hospital, Division of Mental Health and Substance Abuse, Oslo, Norway; 4 Child and Adolescent Mental Health Research Unit, Division of Mental Health and Addiction, Oslo University Hospital, Oslo, Norway

**Keywords:** Depression, Cognitive behavioral therapy, Short-term psychodynamic psychotherapy, Psychoanalytic, Effectiveness, Outpatient clinic

## Abstract

**Background:**

More studies with low risk of bias on the effectiveness of cognitive behavioral therapy (CBT) and short-term psychodynamic psychotherapy (STPP) for major depressive disorder (MDD) are needed. This study compares the outcome of CBT and STPP and examines the improvements in each treatment, focusing on effect sizes, reliable change, dropout rates, and remission rates, using broad inclusion criteria (e.g. participants using antidepressants or with strong suicidal ideation).

**Methods:**

One hundred patients were randomly allocated to CBT or STPP. All patients were offered either 16 weekly sessions followed by 3 monthly booster sessions in CBT, or 28 weekly sessions in STPP. Primary outcome measures were Hamilton Depression Rating Scale (HDRS) and Beck’s Depression Inventory-II (BDI-II). Secondary outcome measures were Work and Social Adjustment Scale (WSAS), Generalized Anxiety Disorder-7 (GAD-7), Global Assessment of Functioning (GAF) and Short Form Health Survey-12 (SF-12).

**Results:**

No significant differences in outcomes were found between the two treatment groups on any of the measures. The within-group effects were large (> 0.8) for the primary outcome measures and moderate to large for the secondary outcome measures. According to the reliable change index (RCI), 79% of patients reliably improved on HDRS and 76% improved on BDI-II, whereas respectively 6% and 10% reliably deteriorated.

**Conclusions:**

These findings support the assumption that CBT and STPP are equally effective treatments for patients with depressive disorders in psychiatric outpatient clinics. Additionally, they strengthen the evidence for the effectiveness of both CBT and STPP in these settings, while also highlighting that not all depressed patients respond to short-term treatment.

**Clinical trial registration:**

Clinical Trial gov. Identifier: NCT03022071. Date of registration: 2016-11-14.

**Supplementary Information:**

The online version contains supplementary material available at 10.1186/s12888-025-06544-6.

## Background

Depression is a common mental disorder, a leading cause of disability and a significant contributor to the global burden of disease [1]. Major depressive disorder (MDD) is expected to rank first in overall disease burden in high income countries by 2030 [2]. The effectiveness of both psychotherapy and antidepressants in treating MDD is firmly established, with evidence suggesting that a combination could be the best option for those with moderate and severe depression [3]. However, more than half of patients receiving therapy do not respond and only about one third remit [4]. The debate regarding which psychotherapeutic approach is most efficient has persisted for decades. Numerous systematic reviews and meta-analyses have demonstrated comparable effects across various bona fide therapies, including cognitive behavioral therapy (CBT) and psychodynamic therapy (PDT) [5–10].

A network meta-analysis by Cuijpers et al. [7] comparing all common psychotherapies for depression in terms of efficacy, acceptability and long-term outcomes, found that all therapies were more effective than care-as-usual and waiting list. However, dropout rates were higher in PDT as compared with care-as-usual, which may indicate that this treatment approach was less acceptable. Of the 331 randomized controlled trials included, only one third were assessed as having a low risk of bias, with 211 studies examining CBT and only 21 examining PDT.

An umbrella review applying the new model for an empirically supported treatment (EST) using the conservative and strict Grading of Recommendations Assessment, Development, and Evaluation (GRADE) system for common mental disorders, concluded that PDT is an empirically supported treatment [8]. PDT was found superior to both active and inactive control groups in reducing depressive symptoms. However, the authors suggest downgrading the evidence for PDT vs. other active therapies for mood disorders by one level (from high to moderate), due to two methodological shortcomings in several studies, i.e., the risk of bias concerning random sequence generation and allocation concealment.

In a comprehensive overview of randomized controlled trials conducted on the effectiveness of PDT in various disorders, Lilliengren [9] identified 298 studies, including 86 studies comparing PDT and CBT. Most studies were conducted on therapies offering 12–40 sessions (63%). Of the included studies, 59 concluded with no significant differences, while 21 favored CBT and 6 favored PDT. Yet only 22.5% of the studies focused on mood disorders. Given the recurrent debate concerning the relative efficacy of PDT to CBT, the fact that about a quarter of all the included comparisons indicated an advantage for CBT, is interesting. Lilliengren notes that some studies supporting CBT have been criticized for misrepresenting PDT, highlighting the necessity of sound study design and balanced research group composition in comparative research.

Several previous studies comparing CBT and PDT for depression have involved highly selective patient groups, included only a small number of participants, or employed methodologies, designs and treatment protocols that differ significantly [11–16]. As a result, existing reviews and meta-analyses typically include studies from a range of clinical settings and timeframes.

To our knowledge, only five studies have examined differences and similarities in outcomes between CBT and short-term psychodynamic therapy (STPP) for MDD in regular clinical outpatient settings. Shapiro et al. [17] included 117 patients with mild to severe depression in a randomized controlled trial (RCT) comparing CBT and psychodynamic-interpersonal psychotherapy (PI). To minimize confounding between therapist characteristics and treatment methods, the same therapists treated patients in both approaches. They found that CBT and PI were equally effective on most measures, irrespective of the severity of depression or the duration of treatment (8 and 16 sessions), except for a small advantage of CBT on the Beck Depression Inventory (BDI). Gibbons et al. [18] found no differences in Hamilton Depression Rating Scale (HDRS) scores between CBT and STPP (16 sessions) in a non-inferiority study including 237 patients with MDD from a community mental health center. However, they could not conclude that STPP was significantly non-inferior to CBT regarding psychosocial functioning or quality of life, as statistical power was only set for testing non-inferiority on the primary outcome measure. Further, they excluded all patients who had suicidal thoughts judged to require more intensive psychotherapy, which may decrease the ecological validity of their findings. Driessen et al. [19] included 341 patients with MDD from three psychiatric outpatient clinics and proved non-inferiority of STPP relative to CBT for HDRS scores, but not for remission (HDRS ≤ 7) rates. The HDRS assessors were not blinded to the treatment condition, which may have increased the risk of concealment bias. Meganck et al. [20] randomized 100 patients with MDD and either self-critical or dependent personality styles to receive 16 to 20 sessions CBT or STPP, with the aim of exploring possible moderator effects. They found no interaction and no evidence for a difference in effectiveness between the treatments. While their study design was rigorous, the results were somewhat weakened by a relatively large dropout rate, with 23 participants receiving four or less therapy sessions. Dos Santos et al. compared the long-term effects of CBT and STPP on depression, measured by the Beck Depression Inventory-II (BDI-II), and on functioning, assessed with the Functioning Assessment Short Test (FAST), three years post-intervention [21]. Their findings showed no significant differences between the groups, and a sustained effect on both depression and functioning among those who completed treatment and participated in the follow-up. These results should be interpreted with caution, as only unmedicated patients without comorbidities or suicide risk were included, and the number of patients who completed therapy and responded at follow-up was reduced to 40 (20 per group) from the initial 243 that were included.

In clinical practice, psychodynamic treatments have traditionally been of longer duration and involved more sessions than CBT. Consequently, some studies have compared the outcomes of longer-term PDT with CBT. A study of patients with depression by Huber et al. [22], which included on average 88 sessions of PDT and 45 sessions of CBT, found no significant differences in depressive symptoms after three years. However, psychoanalytic therapy (PA) outperformed both PDT and CBT. Critics, such as Siegfried Zepf [23], have argued that this may be explained by the fact that PA provided patients with more sessions (mean 234 sessions). Another study by Leuzinger-Bohleber et al. compared long-term CBT (average of 57 sessions) and long-term psychoanalytic therapy (PAT) (average of 234 sessions) for chronically depressed patients, and found no significant differences between the groups after three years [24], but more change in personality structure in the PAT condition compared with the CBT condition at 5-year follow-up [25]. The same challenge with different number of sessions also apply to their study, but the authors argue that this is necessary as the approaches have different conceptualizations of depression, which demands different treatment intensities and durations.

In sum, most studies show no major significant differences between neither longer-term PDT nor STPP compared to CBT in the treatment of depression. However, there is still a scarcity of studies with rigorous design in regular outpatient clinics, and more effectiveness studies with sound methods and lack of bias are still needed.

The current study was designed to meet the limitations of previous studies and is part of the research project Mechanisms of Change in Psychotherapy (MOP), an RCT for patients with MDD at two public outpatient clinics in Norway. Depressed patients were randomly assigned to receive either 19 sessions of CBT within a 28-week period, or 28 weekly sessions of STPP, as described in more detail in the study protocol [26]. Several measures were taken to reduce the risk of bias concerning random sequence generation and allocation concealment, and the research group has a balanced composition with allegiances to both CBT and PDT. In addition, patients receiving antidepressant medication or having suicidal ideation were not excluded. The results from this study may consequently have high ecological validity and low risk of bias.

The main aims of the MOP project were to examine moderators and mediators of change and not differences in overall outcome between CBT and STPP. Our initial assumption was that there would be no significant differences in the outcome measures between the two groups, and the small sample size in the project does not allow for non-inferiority testing. However, on the background of the need for more studies comparing STPP and CBT as documented above, the present study may contribute to fill some important gaps in the current knowledge.

The aim of the present study was to examine the outcome of CBT and STPP in the setting of two public psychiatric outpatient clinics in Norway. The primary outcome was severity of depressive symptoms assessed by the observer-rated HDRS, measured at baseline and after 28 weeks, and the patient-rated BDI-II, measured at baseline and after 8 weeks, 16 weeks and 28 weeks. Secondary outcomes were psychosocial functioning, anxiety and quality of life, measured with Global Assessment of Functioning scale (GAF), Work and Social Adjustment Scale (WSAS), Generalized Anxiety Disorder 7 (GAD-7) and Short Form Health Survey-12 (SF-12). The GAF, SF-12 (MCS) and SF-12 (PCS) were assessed at baseline and after 28 weeks, while WSAS and GAD-7 were assessed at baseline and after 8 weeks, 16 weeks and 28 weeks. More specifically, we investigated the following research questions:

1. Primary outcomes

- What are the effect sizes, both between and within groups, of the primary outcome measures, i.e., observer-rated and self-reported depressive symptoms?
- What are the response and remission rates based on observer-rated and self-reported depressive symptoms?
- What is the proportion of patients showing reliable improvement and deterioration for the primary outcomes?

2. What is the proportion of patients in diagnostic remission?
3. What are the effect sizes, both between and within groups, for the secondary outcomes?
4. What are the dropout rates and are there indications that these rates differ between treatment modalities?

## Methods

### Setting, participants and randomization

Two psychiatric outpatient clinics in Oslo, Nydalen and Vinderen, participated in the MOP study. The clinics are part of the public health care system and require that patients are referred by a psychologist or doctor, often a general practitioner. Both clinics treat patients with a wide range of mental illnesses. Patients pay for sessions until they reach the limit of 230 euros, after which all health care is free of charge.

The study included 100 patients. The sample size was determined by considering both direct and indirect effects [26]. With reference to a t-test comparison of two independent groups as the total effect, a sample size of 100 participants would provide a power of 0.84 (two-sided t-test, significance level of 5%) to detect a difference of a medium (0.6) effect size.

Patients were recruited consecutively as they were referred to the outpatient clinics with depressive symptoms as their main reason for referral. The inclusion criteria were MDD according to the fourth edition of Diagnostic and Statistical Manual of Mental Disorders (DSM-IV) [27], age 18–65 years, an ability to understand, write and speak a Scandinavian language, and willingness to give informed consent. Exclusion criteria were current or past neurological illness, psychotic disorders, traumatic brain injury, bipolar disorder type 1, current alcohol and/or substance dependence disorders, developmental disorders, and intellectual disability.

After baseline assessments, patients were assigned a trial ID and allocated to either CBT or STPP by using computer randomization. Randomization was not stratified, and no block randomization was applied. This procedure was conducted by the principal investigator. Randomization was performed by the generation of a sequence of random numbers that determined to which group the trial participants were allocated. The principal investigator received the treatment allocation (CBT or STPP) from an independent third party located in a separate part of the hospital, ensuring no influence from study personnel. To minimize bias, strict allocation concealment made sure that the person judging eligibility was not aware of the assigned study condition.

### Therapists and steering group

A total of 18 therapists participated in the treatment, comprising 12 women and six men. Among them, there were nine psychologists, six psychiatrists and three psychiatric nurses. All therapists had a minimum of two years of training in CBT or PDT. For this study they all went through one year of training on the principles of CBT and STPP in supervision groups, including a trial case, before receiving patients in the study. The mean number of patients treated by each therapist was 5.6, ranging from one to 12. The average age was 45 years (SD = 8.5), ranging from 31 to 63, with a mean of 47 in the CBT group and 43 in the STPP group. They had a mean of 14 years (SD = 5.5) of experience as therapists, 15 years in the CBT group and 13 in the STPP group.

The steering group of the project consists of two professors (TD and JIR) and one senior researcher (JHE) specialized in CBT, and two professors specialized in PDT (TW and RU). One member of the steering group (KO) has no formal training in any of the psychotherapeutic approaches. They have weekly in-person meetings to oversee the agreed project goals. The three steering group members specialized in CBT supervised the CBT therapists in the project, while the two members specialized in PDT supervised the STPP therapists.

### Treatment interventions

#### Cognitive behavioral therapy

The CBT treatment consisted of 16 weekly sessions followed by three booster sessions at monthly intervals. The treatment was thus offered within a time frame of 28 weeks. Treatment principles were based on «Cognitive Therapy of Depression» by Aaron Beck [28].

The sessions were structured yet flexible. The therapists were active and facilitated collaboration with the patient. The patients were given relevant homework assignments and encouraged to conduct behavioral experiments. Each session typically began with a mood score assessment from 0 to 100, followed by a review of the previous week’s homework assignments, and an agenda was collaboratively set for the current session. At the end of each session, the therapist summarized the content of the session and proposed homework assignments based on the presenting problems and current state of the patient.

Therapists were encouraged to utilize interventions such as Socratic questioning, using the ABC and Diamond model, challenging automatic thoughts, focusing on behavioral activation and identifying thinking traps. Socratic questioning is a technique where therapists use open-ended, probing questions to help clients examine and challenge their thoughts and beliefs, fostering insight and promoting more balanced, adaptive thinking. The ABC model is a framework that explains how an Activating event (A) triggers Beliefs (B), which then lead to Consequences (C), emphasizing the role of beliefs in shaping reactions to events. The Diamond model illustrates how thoughts, emotions, physical sensations, and behaviors are interconnected. Challenging automatic negative thoughts involves identifying and critically examining distorted or unhelpful thoughts to replace them with more realistic and balanced thinking. Behavioral activation is about helping patients identify and engage in positive, rewarding activities to reduce depression by breaking the cycle of avoidance and inactivity. Thinking traps are distorted patterns of thinking that contribute to negative emotions and unhelpful behaviors, such as catastrophizing, overgeneralizing, or black-and-white thinking.

In session 1–3, patient and therapist co-created a list of the patients’ current treatment goals and challenges, as well as a case formulation describing the relationship between past experiences, current dysfunctional beliefs, and response patterns. In addition, the therapists focused on building the therapeutic alliance and socialization to the therapeutic model. In session 4–16, the therapists aimed to alleviate depressive symptoms by using the abovementioned techniques to change patients’ dysfunctional cognitions, assumptions, and attitudes. The booster sessions, 16–19, which were provided by the same therapist, offered patients an opportunity to consolidate the improvement made during therapy, and to address any potential relapse.

#### Short-term psychodynamic psychotherapy

The STPP treatment consisted of 28 weekly sessions. Treatment principles were based on «Long-term psychodynamic psychotherapy» by Glen O. Gabbard [29], which according to the author can be applied to shorter as well as time-limited therapies. This basic text outlines central principles of psychodynamic psychotherapy such as the significance of unconscious mental functioning, the importance of childhood experiences in concert with genetic factors in shaping adult mental life, and how the phenomena transference, countertransference and the patient’s defenses and resistance may affect the therapy process. In the present study, the same time-limited frame of STPP was applied as in two other randomized studies investigating STPP and CBT. Both the RCT by Goodyer et al. comparing STPP, CBT and treatment as usual [30], and the RCT by Ulberg et al. investigating a specific treatment technique in STPP [31], applied a 28-session time frame as described by Cregeen et al. [32].

The STPP therapists aimed to alleviate depressive symptoms by providing new insights into the connections between past and present experiences, focusing on relational challenges, difficult emotions, defense mechanisms and other unconscious material. Guidelines as to how to present the treatment rationale of STPP to the patient, as well as the respective roles of the patient and the therapist, were co-developed by the therapists and the researchers. These included the treatment frame, session frequency, arrangements for holidays and absences from sessions. The therapists were also involved in creating written treatment procedures for this study.

Within the first three sessions, the therapists made a case formulation together with their patients that included symptoms and problems, predisposing and precipitating life events or stressors, and offered suggestions on what could be the maintaining influences of their difficulties. Therapist and patient then agreed on what should be the focus of the treatment. Within session 8–12, the therapists were instructed to make at least one reference to treatment focus and the patient’s perception of the therapy so far. How the themes the patient brought to sessions related to the focus, and potential insights and views about the treatment, should also be mentioned at least once within session 16–20. During this period the therapists were asked to bring up the forthcoming treatment termination at least once. The end of treatment, the patient’s current problems, potential insights and how to make use of such insights after treatment should be major themes during session 20–28.

During treatment, the therapists explored sensitive topics, addressed transactions in the therapeutic relationship, used material about interpersonal relationships outside therapy as the basis for interventions, encouraged exploration of thoughts and feelings about therapy, and interpreted direct manifestations of transference with moderate intensity. In line with Leichsenring and Schauenburg’s [33] suggestions for an evidence-based unified protocol for STPP in depression, these elements were to be applied in a flexible manner adapted to the individual patient’s motivation, severity of pathology and needs.

### Treatment integrity

All therapy sessions were video-recorded and manual fidelity was monitored by experienced supervisors in one-hour weekly (STPP) and two hours bi-weekly (CBT) group supervisions. The supervision focused on the initial phase of treatment, case formulation, individual treatment strategies and termination of therapy.

To ensure that the two therapy approaches differed, a subset of video recordings from randomly selected sessions were evaluated by using the Comparative Psychotherapy Process Scale (CPPS) [34]. An expert panel, comprising both STPP and CBT supervisors, collaborated with the raters to establish a consensus on item interpretation. Two independent raters, blinded to treatment allocation, then each assessed 10 randomly chosen sessions from both CBT and STPP (in total *N* = 40 sessions). To examine the reliability, a subset of sessions (*N* = 10) were assessed by both raters, and reliability was estimated by intraclass correlation (ICC), more specifically ICC (2.1) [35]. The single-measure ICC, employing a consistency definition, indicated good reliability for both the psychodynamic subscale (ICC = 0.94, CI:0.76–0.98) and the cognitive-behavioral subscale (ICC = 0.95, CI:0.82–0.99). The independent raters scored the STPP therapists significantly higher on the psychodynamic subscale than the CBT therapists (2.56 vs. 0.42, *p* < 0.001) and the CBT therapists significantly higher on the cognitive-behavioral subscale than the STPP therapists (3.33 vs. 0.61, *p* < 0.001).

### Diagnostics

Patients were assessed for symptom disorders according to DSM-IV criteria using the Mini International Neuropsychiatric Interview 6.0.0 (MINI) [36]. The MINI was administered both pre- and post-treatment. Personality disorders were assessed using the Structured Clinical Interview for DSM-IV Axis II Personality Disorders (SCID-II) [37].

In DSM-IV, the classification of personality disorders is polythetic. That is, the criteria within each disorder are neither necessary nor sufficient. Psychometrically spoken, the criteria are “causal indicators” gradually creating the construct, rather than “effect indicators” affected by variations of a latent construct [38]. A variable counting the number of fulfilled personality disorder criteria can thus be seen as a severity index. In the current sample, the reliability of this severity index, estimated by ICC (2.1) based on scores from six raters scoring 11 patients, was 0.99 (CI:0.97-1.00).

#### Primary outcomes

**Hamilton depression rating scale**. The level of depression was assessed by an observer using the HDRS at baseline and after 28 weeks [39]. The HDRS is composed of 17 items, each rated on a scale from 0 to 4 or 0 to 2, covering a range of symptoms experienced by the patient within the last week, including depressed mood, guilt, suicidal ideation, insomnia, agitation, and weight loss. The total score on the HDRS provides a measure of the severity of depression, with scores typically categorized as follows: 0–7 (normal), 8–13 (mild depression), 14–18 (moderate depression), 19–22 (severe depression), and ≥ 23 (very severe depression). To examine the reliability of HDRS, we used ICC (2.K) [35]. Four raters scored 10 patients. The reliability coefficient for HDRS was 0.97 (CI:0.92–0.99) for relative decision and 0.96 (CI:0.86–0.99) for absolute decision.

**The Beck depression inventory-II**. The level of depression was also assessed by self-report using BDI-II at baseline, 8 weeks, 16 weeks and 28 weeks [40]. Comprising 21 items, the BDI-II assesses various depressive symptoms experienced during the preceding two weeks, including feelings of sadness, pessimism, guilt, and fatigue, among others. Patients rate the severity of each symptom on a scale ranging from 0 to 3, with higher scores indicating greater severity of depressive symptoms. The total score is calculated by summing the individual item scores, with the following established cut-offs: 0–13 (minimal depression), 14–19 (mild depression), 20–28 (moderate depression), and 29–63 (severe depression). BDI-II has demonstrated excellent psychometric properties in diverse samples [41]. The combination of HDRS and BDI-­II cover many of the domains that are shown to be important to depressed patients [42].

#### Secondary outcomes

**Generalized anxiety disorder 7**. The level of anxiety was measured by self-report using GAD-7 [43]. The GAD-7 consists of seven items that capture common symptoms of anxiety, including excessive worry, restlessness, and difficulty concentrating. Patients rate the frequency of each symptom over the past two weeks on a scale ranging from 0 (not at all) to 3 (nearly every day). Total scores range from 0 to 21, with higher scores indicating greater severity of symptoms. Established cut-offs are as follows: 0–4 (minimal anxiety), 5–9 (mild anxiety), 10–14 (moderate anxiety), and 15–21 (severe anxiety). The GAD-7 has shown excellent internal consistency [44], and strong validity and reliability [45].

**Global assessment of functioning scale.** The level of psychosocial functioning was measured by an observer using GAF [46]. The scores were split into a function score (GAF-F) and symptom score (GAF-S) to measure the level of functioning and symptom severity separately [47]. The GAF-F score encompasses a broad range of functioning, from severe impairment (e.g., inability to maintain personal hygiene) to superior functioning (e.g., stable relationships, high productivity). The GAF-S score encompasses a broad range of symptom severity, from no symptoms to severe symptomatology (e.g. suicide attempts with a clear intention to die). To examine the reliability of GAF-F and GAF-S, we used ICC (2.K) [35]. Four raters scored 11 patients. For GAF-F, the reliability coefficient for relative decision was 0.83 (CI:0.57–0.95) and 0.82 (CI:0.57–0.95) for absolute decision. For GAF-S, the reliability coefficients were 0.92 (CI:0.80–0.98) and 0.90 (CI:0.74–0.97), respectively.

**Work and social adjustment scale.** Functioning was also assessed by self-report using WSAS [48]. WSAS consists of five items that measure impairment in work, social leisure activities, private leisure activities, home management, and close relationships. Patients rate the extent to which their mental health difficulties have interfered with each domain on a scale ranging from 0 (not at all) to 8 (severely). The total score is 40, with higher scores indicating greater impairment. WSAS has been shown to have high internal reliability, and is sensitive to treatment effects [49].

**12-Item short form health survey.** The health-related quality of life was measured by self-report using SF-12 [50]. The SF-12 consists of 12 items that assess eight health domains covering the past four weeks: physical functioning, role limitations due to physical health problems, bodily pain, general health perceptions, vitality, social functioning, role limitations due to emotional problems, and mental health. These items are aggregated to generate two measures: Physical Component Summary (PCS) and the Mental Component Summary (MCS). The SF-12 is a psychometrically sound instrument for measuring health-related quality of life for people with severe mental illness [51].

### Assessments and reliability

As prespecified in the protocol, HDRS, GAF-S, GAF-F, SF-12 (MCS), SF-12 (PCS) were assessed at baseline and after 28 weeks, and BDI-II, WSAS and GAD-7 were assessed at baseline and after 8 weeks, 16 weeks and 28 weeks.

The baseline and follow-up assessments were conducted by a group of clinicians who did not take part in the study treatments. Their training consisted of several sessions in which they reviewed video recordings of previous assessments, and reviews of their own assessments alongside clinical experts in both CBT and STPP. The assessors also received supervision on a regular basis by experienced raters and clinicians, and consensus meetings were held to assure reliability of assessments. To minimize bias, the assessors were blinded to the therapy approach.

### Statistical methods

All statistical analyses were done in SPSS 29.0. We adhered to the intention-to-treat (ITT) principle, including all patients regardless of noncompliance, protocol deviations, or withdrawal. To investigate whether there were indications that the “missing-at-random” assumption was met, we conducted several t-tests and chi-square tests to determine potential differences in baseline variables between patients with missing data and patients without missing data. In total, 81 patients (81%) attended the 28-week evaluation (CBT: 39, STPP: 42). No significant differences were found between those lost to follow-up and those assessed at 28 weeks with respect to demographics (age, gender), treatment variables (previous treatment and antidepressant medication), baseline symptoms (HDRS and BDI-II), or psychosocial functioning (GAF and WSAS). These findings provide support for the assumption that the data are “missing at random”.

Dichotomous data (response rates, remission rates, diagnostic status, and dropout) were analyzed using Chi-Square statistics. Cohen’s d was calculated by using the mean differences between and within groups, divided by the total variance in the model (including residuals, intercept, and slope), following the method described by Westfall et al. [52].

We used the Reliable Change Index (RCI) to calculate the number of patients who were reliably changed on HDRS and BDI-II during treatment [53]. The RCI is an index of change based on the standard error of the difference between two time-points, taking the reliability of the measure into account (RCI = 1.96 x SE_Diff_). For the calculation of RCI for HDRS, we used the inter-rater reliability of HDRS in the present sample, *0.97*, which corresponds to the reliability of 0.96 and 0.98 in two studies of one day test-retest reliability of HDRS [54]. A change of 3.10 represented reliable change. Consequently, patients with decrease in HDRS equal or above 3.10 were regarded as reliably improved, whereas those with an increase in HDRS equal or above 3.10 were regarded as reliably deteriorated. The RCI for BDI-II was based on the pooled seven days test-retest reliability of BDI-II in clinical populations, i.e., *Pearson’s r* = *0.92* reported by Wang & Gorenstein [41]. A pre-post change of 5.88 represented reliable change. Patients with decrease in BDI-II equal or above 5.88 were regarded as reliably improved, and those with an increase in BDI-II equal or above 5.88 as reliably deteriorated.

Linear mixed models were used for all outcome variables [55, 56]. These outcome variables were included as the dependent variables whereas time and the interaction between time and treatment (time*treatment) were included as the independent variables. Time was coded as 0-2-4-7 for variables that were measured at four time points (BDI, WSAS, and GAD-7), which implies that each whole number represents a duration of approximately one month. For outcome variables with two measurement occasions, time was coded as 0–1. All outcome variables were approximately normally distributed for all time points, allowing the use of maximum likelihood estimation.

We adhered to the principles of model building by comparing a simple linear regression model (“baseline model”) against more complex models. The baseline model only included the time variable and intercept as fixed effects. The − 2 Log Likelihood (LLH) and Akaike’s Information Criterion (AIC) were utilized as the primary indices for assessing model fit. The AIC adjusts for model complexity by penalizing the inclusion of additional parameters, making it particularly useful for comparing models of varying complexity. Consequently, the AIC was given the greatest weight when interpreting models relevant to the research hypotheses. Dependency in the longitudinal data was accounted for by including a random intercept and random slope at the individual level, with the autoregressive heterogeneous order I (ARH1) covariance structure for the random effects [56]. However, in those instances where the model did not converge, the covariance structure was simplified to the autoregressive model (AR1). See supplement for details about the model fit of the different models.

To evaluate whether there were differences in outcomes between the two treatment sites, we included site in the third step and the site*time interaction in the fourth step (only primary outcome variables). Similarly, the effect of the number of sessions was evaluated by including an interaction between time and the number of sessions, which was treated as a continuous variable.

For the analysis of the therapist effect (*n* = 18), a third level was added, implying that patients are nested within therapists. This implies the inclusion of a random intercept and random slope at the therapist level. A significant effect for random slope, as well as improved model fit, would imply differences in outcome across therapists.

## Results

### Participant flow and characteristics

**Fig. 1:**
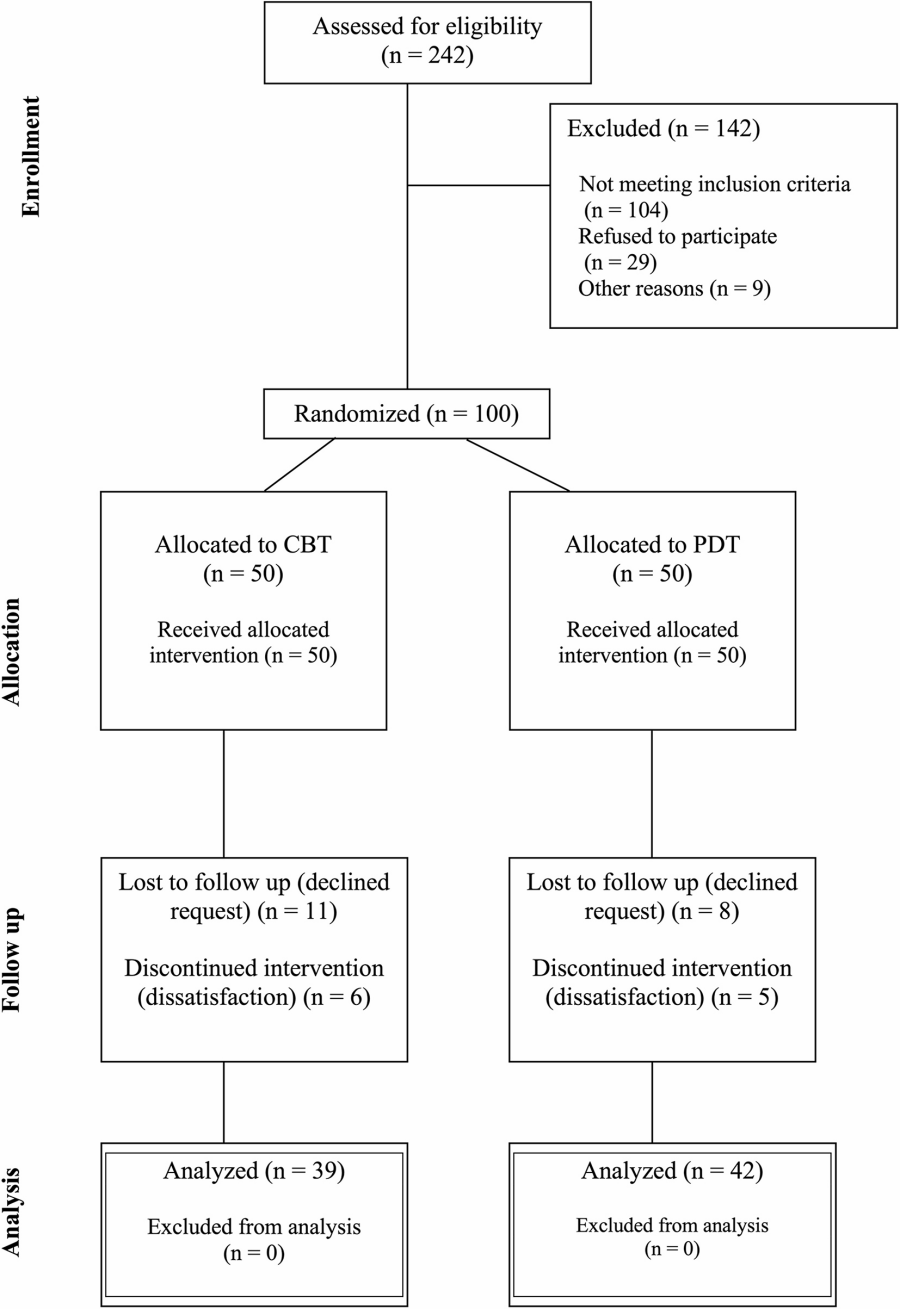
Flowchart of patients’ progress through the phases of the trial

Recruitment occurred from November 2017 to September 2022. In total, 242 patients were assessed for eligibility. Of these, 104 did not meet the inclusion criteria, 29 declined to participate, and nine were excluded for other reasons. After 28 weeks, 11 patients were lost to follow up in the CBT group, and eight were lost in the STPP group. A flow-diagram is presented in Fig. 1. Clinical characteristics and demographic information about participants are shown in Table 1.

**Table 1:** Demographic and clinical characteristics

Baseline characteristics	Total (*N* = 100)	STPP (*N* = 50)	CBT (*N* = 50)
	*n*	*n*	*n*
Age (mean)	31	30	32
Gender			
Female	59	30	29
Male	41	20	21
Antidepressant use	42	18	24
Psychotherapy experience ^a^	62	29	33
Previous admissions ^b^	7	4	3
Ethnicity			
European	96	50	46
Other	4	0	4
Marital status			
Married/partner	39	20	19
Unmarried/no partner	61	30	31
Education level ^c^			
Very low (< 3 years)	8	3	5
Low (*≥ 3 years)*	29	14	15
Intermediate (*≥ 6 years)*	45	19	26
High (*≥ 9 years)*	18	14	4
Job status			
Working	68	31	37
Student	22	12	10
Social security benefits	10	7	3
Axis 1 disorders			
Major depressive disorder	100	50	50
Recurrent depressive disorder	67	32	35
Bipolar II disorder	1	1	0
Dysthymia	1	1	0
Panic disorder	13	6	7
Panic disorder w/agoraphobia	14	7	7
Agoraphobia	9	5	4
Generalized anxiety disorder	4	2	2
Social phobia	15	7	8
Obsessive-compulsive disorder	1	0	1
Personality disorders			
Avoidant	15	9	6
Dependent	1	0	1
Obsessive-compulsive	1	1	0
Paranoid	5	2	3
Personality disorder NOS	6	3	3

Note. Percentages are not provided as the total number of patients is 100

^a^ Previous psychotherapy experience (> 1 x week). ^b^ Previous admission in psychiatric hospital. ^c^ Education after primary school (ten years). NOS = Not otherwise specified

### Primary outcomes (HDRS and BDI-II)

#### Effect sizes

There were no significant differences in HDRS between CBT and STPP at 28 weeks, as the time*treatment interaction was not significant (*p* = 0.283) (Table 2). Accordingly, the between-group effect size was small (d < 0.3) (Table 3). The effect of time was highly significant (*p* < 0.001) (Table 2), and the within-group effect sizes were large (d > 0.80).

**Table 2:** Results from linear mixed model analyses

Dependent variable	Estimate	SE	CI (95%)	t	*p*
BDI-II ^a c^					
Time	–1.60	0.23	–2.04 to − 1.15	–7.1	< 0.001
Time x treatment	–0.12	0.32	–0.76 to 0.53	–0.4	0.720
HDRS ^a d^					
Time	–6.93	0.97	–8.86 to − 5.01	–7.2	< 0.001
Time x treatment	–1.50	1.39	–4.25 to 1.26	–1.1	0.283
GAF-S ^b d^					
Time	9.61	1.37	6.89 to 12.32	7.0	< 0.001
Time x treatment	0.82	1.96	–3.09 to 4.73	0.4	0.677
GAF-F ^b d^					
Time	11.63	1.38	8.90 to 14.37	8.5	< 0.001
Time x treatment	0.15	1.98	–3.79 to 4.09	0.1	0.938
WSAS ^b c^					
Time	–1.38	0.20	–1.78 to − 0.98	–6.9	< 0.001
Time x treatment	0.38	0.29	–0.19 to 0.94	1.3	0.192
SF-12 (MCS) ^b d^					
Time	8.56	1.95	4.71 to 12.41	4.4	< 0.001
Time x treatment	–0.48	2.78	–5.96 to 5.00	–0.2	0.863
SF-12 (PCS) ^b d^					
Time	–0.58	1.43	–3.43 to 2.27	–0.4	0.689
Time x treatment	0.02	2.05	–4.04 to 4.09	0.0	0.991
GAD-7 ^b c^					
Time	–0.50	0.11	–0.72 to − 0.28	–4.5	< 0.001
Time x treatment	–0.11	0.16	–0.43 to 0.20	–0.7	0.481

^a^ Primary outcome. ^b^ Secondary Outcome. ^c^ Time points 0, 2, 4 and 7 months. ^d^ Time points 0 and 7 months. HDRS = Hamilton Depression Rating Scale; BDI-II = Beck Depression Inventory II; GAF-S = Global Assessment of Functioning, Symptom Score; GAF-F = Global Assessment of Functioning, Functioning Score; WSAS = Work and Social Adjustment Scale; SF-12 (MCS) = Short Form Health Survey, Mental Health Component Scale; SF-12 (PCS) = Short Form Health Survey, Physical Component Scale; GAD-7 = Generalized Anxiety Disorder-7

**Table 3:** Means of baseline and 28-week measures for cognitive behavioral therapy (CBT) and short-term psychodynamic psychotherapy (STPP). Effect sizes (Cohens d) for within-group differences and between-group differences

Measure	Baseline		28 weeks		Cohens d		
	STPP	CBT	STPP	CBT	*STPP*	*CBT*	*Between groups*
HDRS ^a^	17.9 (5.5)	18.3 (5.8)	11.0 (5.2)	9.9 (5.0)	1.23	1.50	0.27
BDI-II ^a^	26.5 (7.6)	28.5 (7.5)	14.9 (9.4)	15.7 (10.7)	1.34	1.48	0.14
WSAS ^b^	26.2 (6.3)	25.2 (6.9)	15.6 (9.7)	17.9 (9.8)	1.24	0.85	0.39
GAF-S ^b^	52.4 (4.9)	53.0 (3.2)	62.0 (8.6)	63.5 (7.7)	1.18	1.29	0.11
GAF-F ^b^	56.2 (6.4)	56.5 (4.7)	67.6 (9.8)	67.9 (8.9)	1.23	1.23	0.00
GAD-7 ^b^	13.2 (4.5)	13.5 (4.1)	9.7 (5.2)	9.2 (5.1)	0.72	0.90	0.17
SF-12 (MCS) ^b^	28.9(10.7)	30.4 (9.8)	37.5 (7.5)	38.5 (8.7)	0.93	0.88	0.05
SF-12 (PCS) ^b^	45.9 (7.6)	44.7 (8.7)	45.2 (7.7)	44.0 (8.5)	0.09	0.09	0.00

Note. Standard deviations for mean values are presented in parentheses. ^a^ Primary outcome. ^b^ Secondary Outcome. HDRS = Hamilton Depression Rating Scale; BDI-II = Beck Depression Inventory II; GAF-S = Global Assessment of Functioning, Symptom Score; GAF-F = Global Assessment of Functioning, Functioning Score; WSAS = Work and Social Adjustment Scale; SF-12 (MCS) = Short Form Health Survey, Mental Health Component Scale; SF-12 (PCS) = Short Form Health Survey, Physical Component Scale; GAD-7 = Generalized Anxiety Disorder-7

**Fig. 2:**
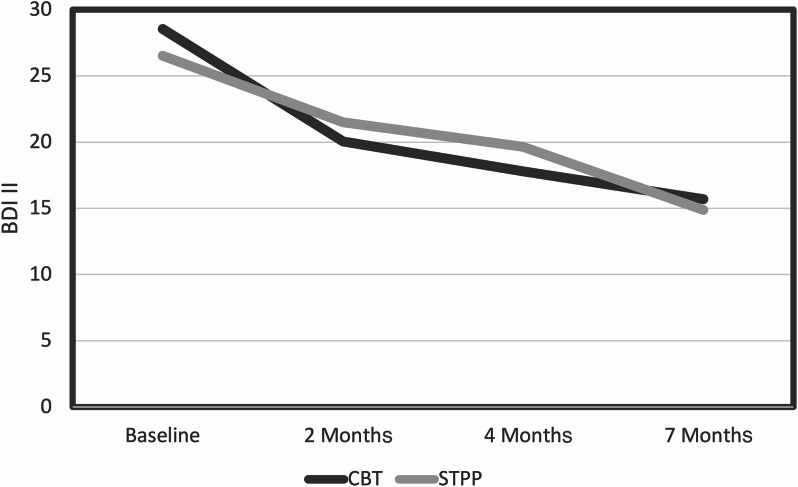
Measures of Beck depression inventory II (BDI-II) over time (months) for patients in cognitive behavioral therapy (CBT) and short-term psychodynamic therapy (STPP)

Similarly, there were no significant differences between groups in BDI-II after 28 weeks, indicated by a non-significant time*treatment interaction (*p* = 0.720), and a small effect size between groups (d < 0.2). The effect of time was highly significant (*p* < 0.001), and the within-group effect sizes were large (d > 0.80). Fig. 2 illustrates the significant reduction in depressive symptoms observed in both groups on BDI-II. The graphs show comparable rates of change between the two interventions, with consistent reductions in BDI-II scores across all time intervals.

#### Symptomatic response and remission rates

Response to treatment in HDRS, defined as a reduction of ≥ 50% from baseline to 28 weeks, was achieved by 42% of the patients (34 out of 81) (Table 4). The Odds Ratio (OR), comparing the odds of achieving treatment response between the two groups, was 0.59 (CI: 0.24–1.42), indicating no statistically significant difference (χ² = 1.40, *p* = 0.24) (Table 4). The proportion of patients in remission, defined as HDRS ≤ 7, was 32% (26 out of 81). The OR for remission was 0.71 (CI: 0.28–1.82), which was not statistically significant (χ² = 0.50, *p* = 0.48).

**Table 4:** Patients not fulfilling criteria for MDD, in remission (HDRS ≤ 7/BDI-II ≤ 9) or responding (HDRS/BDI-II reduction ≥ 50%), and Reliable Change (RCI) for HDRS and BDI-II after 28 weeks

	CBT (*N* = 39) (%)	STPP (*N* = 42) (%)	χ^2^ (*p*)	Odds ratio (CI)
No MDD	31 (80)	30 (71)	0.71 (0.40)	1.55 (0.56, 4.32)
HDRS ≤ 7	14 (36)	12 (29)	0.50 (0.48)	0.71 (0.28, 1.82)
HDRS ≥ 50%	19 (49)	15 (36)	1.40 (0.24)	0.59 (0.24, 1.42)
HDRS RCI Improved	31 (80)	33 (79)	0.01 (0.92)	0.95 (0.32, 2.76)
HDRS RCI Deteriorated	2 (5)	3 (7)	0.14 (1.00)	1.42 (0.23, 9.00)
BDI-II ≤ 9	12 (31)	15 (37)	0.30 (0.58)	0.77 (0.30, 1.95)
BDI-II ≥ 50%	20 (51)	22 (54)	0.05 (0.83)	0.91 (0.38, 2.19)
BDI-II RCI Improved	31 (79)	31 (74)	0.17 (0.68)	0.80 (0.28, 2.30)
BDI-II RCI Deteriorated	4 (10)	4 (10)	0.01 (1.00)	0.95 (0.22, 4.08)

Note. Percentages, p-values and confidence intervals are presented in parentheses. MDD = major depressive disorder. HDRS = Hamilton Depression Rating Scale; BDI-II = Beck Depression Inventory II

Response to treatment in BDI-II, defined as a reduction of ≥ 50% from baseline to 28 weeks, was achieved by 52.5% of patients (42 out of 80). The discrepancy between the number of patients in the BDI-II and HDRS (80 vs. 81) is due to one patient not returning the self-completed forms. The Odds Ratio (OR) was 0.91 (CI: 0.38–2.19), indicating no significant difference between the groups (χ² = 0.05, *p* = 0.83). The proportion of patients in remission, defined as BDI-II ≤ 9, was 36.6% (27 out of 80). The OR for remission was 0.77 (CI: 0.30–1.95), with no significant between-group difference (χ² = 0.30, *p* = 0.58).

#### Reliable change and deterioration

In the HDRS, 79% of patients showed reliable improvement, while 6% (*n* = 5) experienced reliable deterioration. There were no statistically significant differences between the groups in rates of reliable improvement (χ² = 0.01, *p* = 0.92), with an OR of 0.95 (CI: 0.32–2.76). Similarly, no significant difference was found in reliable deterioration (χ² = 0.14, *p* = 1.00), with an OR of 1.42 (CI: 0.23–9.00) (Table 4).

For the BDI-II, 76% of patients were reliably improved, and 10% (*n* = 8) showed reliable deterioration. No significant between-group differences were observed for reliable improvement (χ² = 0.17, *p* = 0.68), with an Odds Ratio (OR) of 0.80 (CI: 0.28–2.30). Similarly, no significant difference was found for reliable deterioration (χ² = 0.01, *p* = 1.00), with an OR of 0.95 (CI: 0.22–4.08).

#### Diagnostic remission according to MINI

Of the 81 patients who attended the 28-week evaluation, 61 (75%) no longer met the criteria for MDD and were considered in diagnostic remission, with an OR of 1.55 (CI: 0.56–4.32) (Table 4). There was no significant difference between the two treatment groups (χ² = 0.71, *p* = 0.40).

### Secondary outcomes (GAF, WSAS, GAD-7 and SF-12)

#### Effect sizes

There were no significant differences between CBT and STPP on any of the secondary outcome measures, as none of the time*treatment interactions were significant (Table 2). All between-group effect sizes were small (d < 0.2), except for WSAS (d = 0.39) (Table 3). In contrast, the effect of time was highly significant (*p* < 0.001) for all measures, except for SF-12 (PCS) (*p* = 0.689) (Table 2). Most within-group effect sizes were large (d > 0.8), except for GAD-7 in STPP (d = 0.72) and SF-12 (PCS) in both groups (d < 0.2).

#### Treatment dropout

Dropout was defined as attending fewer than eight therapy sessions. On average, patients in the CBT group attended 14.9 (SD = 5.2) sessions, while those in the STPP group attended 22.5 (SD = 7.7) sessions. A total of 11 patients dropped out of therapy, including six from the CBT group and five from the STPP group, resulting in an OR of 0.82 (CI: 0.23–2.87). There were no significant differences in dropout rates between the groups (χ² = 0.10, *p* = 0.75).

#### Number of sessions, treatment sites, and therapist effects

The interaction between time and the number of sessions was non-significant for both primary outcomes (HDRS: *p* = 0.577, F = 0.31; BDI-II: *p* = 0.757, F = 0.096). This indicates that the amount of therapy delivered had no overall effect on outcomes. This finding was further supported by the lack of improvement of the model (see Supplementary materials for details on model building and testing).

We also examined whether one treatment site produced better results than the other. The time × site interaction was not significant for BDI-II (*p* = 0.169, F = 1.93), but significant for HDRS (*p* = 0.032, F = 4.7). The estimated difference in change of HDRS across the two sites was 3.4 points (SD: 1.6).

Finally, to assess whether there were differences in change trajectories across therapists, we included random slope for therapists and random intercept for patients. We then tested the significance of the slope variance for the therapist effect. For HDRS, the model did not converge. For BDI-II, the slope variance was non-significant (*p* = 0.686), indicating no significant differences in outcomes across therapists.

## Discussion

There were no statistically significant differences between CBT and STPP in any of the outcome measures. However, we found that both CBT and STPP are associated with an improvement in depressive symptoms and increase in psychosocial functioning and mental health-related quality of life. This supports CBT and STPP as evidence-based treatments for MDD in regular outpatient settings. However, while most patients showed reliable improvement, many did not achieve symptomatic remission, and some even deteriorated. The following discussion will address the comparability of these results and their potential implications.

Regarding primary outcomes, the treatment effects on depressive symptoms and remission rates are comparable to other head-to-head comparisons between CBT and STPP. The patients in the present study improved about eight points on HDRS, which is more than the 5-point improvement in Gibbons et al. [18] and equal to the 8-point improvement found by Driessen et al. [19] in their study. Both studies have treatment protocols that are similar to ours, but the treatment course in the present study is longer (16 vs. 28 sessions in STPP and 16 vs. 19 in CBT). At post-treatment, our patients were less depressed than patients in both the study by Gibbons et al. (HDRS of 10 versus 15) and Driessen et al. (HDRS of 10 versus 16). This may be partly explained by the fact that our patients are also less depressed at baseline. Compared to Meganck et al. [20], the results of the current study were very similar to their pre-treatment HDRS score of 18.2 and reduction to 11.3 after 6 months.

The remission rate in the study by Driessen et al. [19] was 22.7% when defined as HDRS ≤ 7, which again is somewhat lower than in the current study (32%). It might be that our larger proportion of patients in remission is because our patients are less severely depressed at baseline. Driessen et al. [19] speculate that their low remission rate may be related to the relatively low socioeconomic status and income levels of their patients. Comparing socioeconomic factors across studies poses a challenge, but it is not obvious that our patients differ significantly from their sample. Driessen et al. also argue that the low remission rates observed in their study could indicate that extending the treatment courses is necessary to get better outcomes. As our treatment courses are longer and with better results, this may be taken as support to this claim. However, we did not find any evidence that the number of sessions was related to outcome. The present results are in line with those in the study by Meganck et al. [20], where 31.2% of participants were in remission by the criteria of HDRS ≤ 7 after being offered 16 sessions of CBT or STPP. Together these results raise some important questions about the cost-effectiveness of additional sessions. Findings from our one- and three-year follow-ups will provide insights into long-term outcomes, including consideration of cost-effectiveness. Of note, however, all these studies concern short-term therapies, and as indicated in the review by Bone et al. [57], different patients may respond to therapy at different rates, indicating that longer duration of treatment could help more patients.

When defining response as a reduction in HDRS ≥ 50%, the response rate in our sample was 42% (CBT: 49%, STPP: 36%). Again, this is somewhat higher than that in the study by Driessen et al. [19], who found a total response rate of 38% (CBT: 39%, STPP: 37%). The response rate in the current study is also considerably higher than in the study by Gibbons et al. [18] in both CBT (22%) and STPP (16%). These differences in response rates may also be a result of the factors mentioned above. Furthermore, nearly half of their study population consisted of patients with a minority background, primarily African American, whereas in our dataset, the minority population was only 4%. This may also contribute, as being in a minority group is a known risk factor of non-response in psychotherapy [57].

Regarding secondary outcomes, Driessen et al. found no significant differences between CBT and STPP in either anxiety or quality of life [58], or in therapist-rated psychosocial functioning [59]. As they found that the therapist ratings were influenced by therapist characteristics, they called for more research including blinded observer-rated outcome measures concerning psychosocial functioning. The present study provides observer-rated GAF-F scores with high reliability and show improvements in GAF-F scores from approximately 56 to 68, which align closely with the findings reported by Driessen et al. [59].

The dropout rate in the current study is low. According to a meta-analysis of RCTs by Cooper & Conklin [60], the mean dropout rate in treatment of MDD is 17.5% They define dropout as unexpected patient attrition among individuals who were randomized to a treatment, but failed to complete it. We defined dropout as attending less than eight sessions of therapy, with a dropout rate of 11% (CBT: 12%, STPP: 10%). This is significantly lower than that reported by Driessen et al. (31% in CBT and 26% in STPP) with a similar definition of dropout [19]. It is also lower than in the study by Meganck et al. [20], where 23% of patients dropped out before receiving four or less therapy sessions (CBT: 9%, STPP: 14%). It is difficult to speculate whether differences in setting, therapists or research design may play a role in explaining differences in dropout rates across studies, and neither Driessen et al. [19] or Meganck et al. [20] provide any explanation to their large dropout rates.

In a study by van Dijk et al. [61] on risk factors for dropout in a large outpatient sample with depressed patients, they found that higher socio-economic status, higher severity of the depression, comorbid personality disorders, or higher levels of anxiety symptoms, were associated with lower risk of dropout in the treatment of depression. The patients in Driessen et al. [19] were more depressed at baseline, which would suggest a lower chance of dropout. Unfortunately, it is difficult to directly compare socioeconomic factors across studies, and neither Driessen et al. [19] or Meganck et al. [20] provide information on personality disorders.

In line with several other studies of depression, a large proportion of the patients did not respond to treatment or had residual symptoms. As the long-term prognosis of depression concerning relapse and recurrence is still poor [3], and this appears to correlate with the persistence of significant residual symptoms [62], there is an urgent need for even more effective treatment. The present study adds additional support to the assertion that CBT and STPP are effective, but also demonstrate that not all patients remit from short-term treatments, while some even deteriorate. This prompts the question of how we can enhance the effectiveness of psychotherapy even further. Subsequent research should prioritize investigating critical factors, such as identifying moderators and mediators of change to address the crucial question of what therapeutic approaches work for whom, and under what circumstances. Our results suggest that some patients may benefit from being offered alternative therapy formats, additional medications, or non-pharmacological biological treatments.

### Strengths and limitations

The study was conducted in two outpatient clinics within the public mental health services. In contrast to previous studies, we have included patients with current suicidal ideation and patients using antidepressants. Thus, our results may be generalizable to regular outpatient settings. The risk of allegiance bias is lowered by the fact that the study steering group consists of both CBT and STPP therapists. In addition, our randomized study design with random sequence generation, allocation concealment and good psychometric inter-rater reliability further reduced the risk of bias. In addition, we have included several measures both during and after therapy that are seldom reported in effectiveness studies.

However, this study also has several limitations. The relatively small number of patients is a major limitation as we are not able to state non-inferiority of STPP. The MOP study was mainly designed to answer other research questions, such as identifying moderators and mediators of change. For the same reason, no untreated control group was included. Another aspect that may be perceived as a limitation is the disparity in the number of sessions provided, with CBT offering 16 weekly sessions and 3 monthly booster sessions, and STPP offering 28 weekly sessions. We decided to have a dissimilar number of sessions to respect the fact that psychodynamic therapies are often longer and with more sessions than cognitive therapies in regular outpatient clinics. Furthermore, our statistical analyses showed that the number of sessions provided was not associated with outcome. The different number of sessions in the treatment groups may increase the external validity of the study and make the results more applicable to a regular outpatient setting.

The therapists received regular supervision using video-recorded sessions to make sure they provided therapy of high quality and adhered to the treatment protocol. However, such supervision is rarely implemented in routine clinical practice, which could be considered a limitation of the study. Additionally, it should be acknowledged as a limitation that some patients started using antidepressant medication during therapy, which may have further contributed to their improvement. While maintaining stable medication regimens is typically recommended in psychotherapy studies, such restrictions can compromise the ecological validity of the results, which was a priority in the study design.

## Conclusions

The present study revealed no statistically significant differences between STPP and CBT in any of the outcome measures. Both treatments were associated with large effect sizes. With wide inclusion criteria, this study adds to the current research by providing results with high ecological validity. In addition, our findings add further support to the evidence that STPP compares well to CBT in improving depressive symptoms, psychosocial functioning, anxiety, and mental health-related quality of life in regular psychiatric outpatient clinics. However, the findings also demonstrate that not all patients benefit from short-term treatment.

## Electronic supplementary material

Below is the link to the electronic supplementary material.

Supplementary Material 1

## Data Availability

The datasets used and analyzed during the current study are available from the corresponding author on reasonable request.
