# Supplementary material for: Comparative effectiveness of short-term psychodynamic psychotherapy and cognitive behavioral therapy for major depression in psychiatric outpatient clinics: a randomized controlled trial
Source: BMC Psychiatry. 2025 Feb 11;25:113. doi: 10.1186/s12888-025-06544-6 (PMC11817821; doi:10.1186/s12888-025-06544-6)
Supplement: Supplementary file 1 — Supplementary Material 1 [file 12888_2025_6544_MOESM1_ESM.docx]

**SUPPLEMENT**

**Model fit – primary outcomes measures**

We adhered to the principles of model building by comparing a simple longitudinal regression model (“baseline model”) against more complex models. The baseline model included two fixed effects, i.e., time and intercept. As can be seen in Supplement table 1, the LLH and AIC for BDI were 2435.7 and 2441.7 respectively. The first step implicated the inclusion of a random intercept, resulting in substantial better model fit as indicated by a decrease of LLH of 78.5 points and a decrease of AIC of 76.3 points. The variance of the random intercept was significant (p<0.001), indicating large differences in BDI baseline scores across patients. In the second step, a random slope was added. Model fit improved slightly, and the random slope effect was significant at the α < .05 level (p=0.025), implying that change trajectories were significantly different across patients. The third step involved the inclusion of the time*treatment interaction, which did not result in improvement of model fit; LLH did not change whereas AIC went slightly up. Since this model is related to the main research question of the study, it is considered as the final model and presented in more detail in the results section.

For HDRS, the model fit showed a slight improvement in the first step but no improvement in the second step. In the third and fourth steps, the model fit slightly deteriorated when accounting for model complexity, as indicated by an increase in AIC—by two points in the third step and one point in the fourth step. Of note, the model using ARH1 failed to converge from the second step onward. Consequently, the covariance structure was simplified to AR1, which explains the addition of only one parameter at step 2, compared to the two parameters added for BDI at the same step.

**Model fit – secondary outcomes measures**

For GAF-S, model fit improved considerably when the random slope was included (step 2) but did not improve for the other steps (Supplemental table 2). For GAF-F, the pattern was slightly different since model fit improved for both the first and second step. This pattern was the same for WSAS, i.e., improvement of model fit by including random intercept (step 1) and random slope (step 2), but no improvement by including the fixed effect “intervention” (step 3) and “intervention*time” (step 4).

For WSAS, the variance of the random intercept was significant (p=0.003), and the p-value of the slope variance was 0.003, indicating significant differences in change rates across patients over the four measurement points.

For SF-12 (PCS), model fit did only improve for the first step (Supplemental table 3). Thus, the inclusion of random slope, intervention, or intervention*time had no effect on model fit.

SF-12 (MCS) was the next variable that was evaluated. The model with a random intercept failed to converge after 500 iterations. Various covariance structures were tested, including Variance Components, Unstructured, Heterogeneous First-Order Autoregressive, and Compound Symmetry, but none proved successful. Additionally, attempting a random slope without a random intercept also failed to converge. Consequently, both the random slope and random intercept were excluded from the model. Neither the addition of the intervention at baseline (step 3) nor the inclusion of the interaction term (intervention × time, step 4) led to an improvement in model fit.

For GAD-7, model fit had a large improvement by including the random intercept, indicating that there were large differences at baseline of self-reported anxiety symptoms across patients. However, model fit did not improve by including a random slope (p=0.12 for the random slope effect). Thus, there were no significant differences in change rates of self-reported anxiety across patients. By including the time*treatment interaction, model fit deteriorated according to AIC.

**Model fit – additional analyses**

The effect of site assesses whether the one treatment site had better average treatment outcome than the other treatment site. The addition of site at baseline did not change model fit for BDI -II (AIC increased from 2362.4 to 2363.9), suggesting that there were no differences in symptom severity across the two sites. The addition of random slope neither resulted in improved model fit. The HDRS analyses gave a somewhat different picture since AIC went down with 2.5 points after inclusion of the site*time interaction. This model is presented in more detail in the results section.

Including the number of sessions in the model instead of intervention did not provide better model fit, neither for BDI-II nor for HDRS.

To assess the potential differential effect of therapists, the first step of this model should be compared with the baseline model. The random intercept was not included in this analysis, as we assumed by design that there would be no significant differences in BDI-II scores at baseline across therapists. Specifically, therapists were not assigned patients with systematically more severe depressive symptoms than others. It appeared that model fit did not improve by including the random slope. To verify this finding, we introduced a random intercept for patients in the subsequent step, which was compared with step 1 of the main analysis. The AIC increased by 2.6 points, from 2365.4 to 2367.0, indicating that the model fit did not improve. For HDRS, the model with random slope for therapists did not converge after 500 iterations and testing different covariance structures.

**Supplement table 1. Model fit indices for primary outcomes**

|  | BDI | | Nr. | HDRS | | Nr. |
| --- | --- | --- | --- | --- | --- | --- |
|  | LLH | AIC |  | LLH | AIC |  |
| **Effect of intervention** |  |  |  |  |  |  |
| Fixed intercept and time (Baseline model). | 2435.7 | 2441.7 | 3 | 1121.5 | 1127.5 | 3 |
| Step 1. Addition of random intercept | 2357.2 | 2365.4 | 4 | 1116.4 | 1124.4 | 4 |
| Step 2. Addition of random slope | 2350.4 | 2362.4 | 6 | 1115.5 | 1125.5 | 5 |
| Step 3. Addition of intervention | 2350.5 | 2364.4 | 7 | 1115.4 | 1127.4 | 6 |
| Step 4. Addition intervention*time (Final model) | 2350.2 | 2366.2 | 8 | 1114.2 | 1128.2 | 7 |
| **Effect of site** |  |  |  |  |  |  |
| Step 3. Addition of site | 2349.9 | 2363.9 | 7 | 1115.0 | 1127.0 | 6 |
| Step 4. Addition site*time | 2348.0 | 2364.0 | 8 | 1110.4 | 1124.4 | 7 |
| **Effect of number of sessions** |  |  |  |  |  |  |
| Step 3. Addition of number of sessions | 2347.8 | 2361.8 | 7 | 1115.4 | 1127.4 | 6 |
| Step 4. Addition sessions*time | 2347.7 | 2363.7 | 8 | 1115.1 | 1129.1 | 7 |
| **Effect of therapists** |  |  |  |  |  |  |
| Step 1 Addition of random slope for therapists | 2433.2 | 2441.2 | 4 | –* | –* | – |
| Step 2 Addition of random intercept for patients | 2357.0 | 2367.0 | 5 | –* | –* | – |

*Note*: *The model did not converge after 500 iterations and testing different covariance structures.

**Supplement table 2. Fit indices, secondary outcomes; GAF and psychosocial functioning**

|  | GAF-S | | | GAF-F | | | WSAS | | |
| --- | --- | --- | --- | --- | --- | --- | --- | --- | --- |
|  | LLH | AIC | Nr. | LLH | AIC | Nr. | LLH | AIC | Nr. |
| Fixed intercept and time (Baseline model) | 1177.5 | 1183.5 | 3 | 1239.7 | 1245.7 | 3 | 2349.6 | 2355.6 | 3 |
| Step 1. Addition of random intercept | 1177.4 | 1185.4 | 4 | 1231.7 | 1239.7 | 4 | 2277.7 | 2285.7 | 4 |
| Step 2. Addition of random slope | 1136.6 | 1146.6 | 5 | 1206.2 | 1216.2 | 5 | 2259.9 | 2271.9 | 6 |
| Step 3. Addition of intervention | 1135.5 | 1147.5 | 6 | 1206.1 | 1218.1 | 6 | 2259.8 | 2273.8 | 7 |
| Step 4. Addition intervention*time (Final model) | 1135.3 | 1149.3 | 7 | 1206.1 | 1220.1 | 7 | 2258.1 | 2274.1 | 8 |

**Supplement table 3. Model fit indices for secondary outcomes; SF-12 and GAD-7**

|  | SF-12-Ph | | | SF-12-M | | | GAD-7 | | |
| --- | --- | --- | --- | --- | --- | --- | --- | --- | --- |
|  | LLH | AIC | Nr. | LLH | AIC | Nr. | LLH | AIC | Nr. |
| Fixed intercept and time (Baseline model) | 1260.7 | 1266.7 | 3 | 1312.9 | 1318.9 | 3 | 2019.3 | 2025.3 | 3 |
| Step 1. Addition of random intercept | 1252.7 | 1260.7 | 4 | –* | –* | – | 1921.4 | 1929.4 | 4 |
| Step 2. Addition of random slope | 1252.7 | 1262.7 | 5 | –* | –* | – | 1918.4 | 1930.4 | 6 |
| Step 3. Addition of intervention | 1251.9 | 1263.9 | 6 | 1312.0 | 1320.0 | 4 | 1918.1 | 1932.1 | 7 |
| Step 4. Addition intervention*time (Final model) | 1251.9 | 1265.9 | 7 | 1312.0 | 1322.0 | 5 | 1917.6 | 1933.6 | 8 |

*Note*: *The model did not converge after 500 iterations and testing different covariance structures.
